# (*m*-Phenyl­enedimethyl­ene)diammonium *p*-nitro­phenyl­phosphate perchlorate

**DOI:** 10.1107/S1600536807068638

**Published:** 2008-01-04

**Authors:** Yohannes T. Tesema, Teshome B. Yisgedu, Ray J. Butcher, Yilma Gultneh, Bijan Ahvazi

**Affiliations:** a Department of Chemistry, Howard University, 525 College Street NW, Washington, DC 20059, USA

## Abstract

The title compound, C_8_H_14_N_2_^2+^·C_12_H_8_N_2_O_8_P^−^·ClO_4_^−^, was formed by the reaction of α,α-bis-*m*-xylenediamine and sodium bis-*p*-nitro­phenyl­phosphate in the presence of Zn(ClO_4_)·6H_2_O in methanol solution. The two amine groups of the *m*-xylenediammonium ion are each protonated and each hydrogen-bonded to two O atoms of the phosphate anion, which acts as a 1,3-bridge. The ammonium groups are arranged matched face to face and each pair is doubly bridged by two perchlorate ions through hydrogen bonding. In addition, there are also weak C—H⋯O inter­actions. Both the N—H⋯O and C—H⋯O inter­actions are contained in a channel down the *a* axis. The perchlorate oxygen atoms are disordered over two positions with site occupancy factors of *ca* 0.7 and 0.3.

## Related literature

For related literature, see: Gultneh *et al.* (1996 ▶, 1999 ▶)
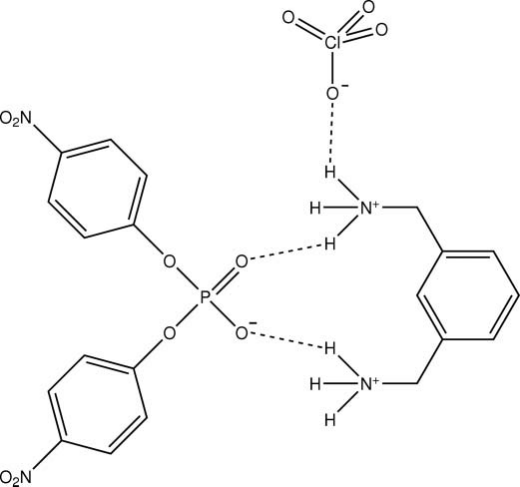


## Experimental

### 

#### Crystal data

C_8_H_14_N_2_^2+^·C_12_H_8_N_2_O_8_P^−^·ClO_4_^−^
*M* *_r_* = 576.84
Triclinic, 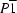

*a* = 8.337 (2) Å
*b* = 11.623 (3) Å
*c* = 13.535 (3) Å
α = 91.22 (1)°
β = 94.32 (1)°
γ = 106.06 (1)°
*V* = 1255.6 (5) Å^3^
*Z* = 2
Mo *K*α radiation
μ = 0.29 mm^−1^
*T* = 293 (2) K
0.35 × 0.29 × 0.17 mm

#### Data collection

Bruker P4S diffractometer
Absorption correction: ψ scan (North *et al.*, 1968 ▶) *T*_min_ = 0.876, *T*_max_ = 0.941
3673 measured reflections
3388 independent reflections
2390 reflections with *I* > 2σ(*I*)
*R*_int_ = 0.026
θ_max_ = 23.0°
3 standard reflections every 97 reflections intensity decay: <2%

#### Refinement

*R*[*F* ^2^ > 2σ(*F* ^2^)] = 0.058
*wR*(*F* ^2^) = 0.154
*S* = 1.02
3388 reflections
383 parameters
92 restraints
H-atom parameters constrained
Δρ_max_ = 0.40 e Å^−3^
Δρ_min_ = −0.28 e Å^−3^

### 

Data collection: *XSCANS* (Bruker, 1997 ▶); cell refinement: *XSCANS*; data reduction: *XSCANS*; program(s) used to solve structure: *SHELXS97* (Sheldrick, 2008 ▶); program(s) used to refine structure: *SHELXL97* (Sheldrick, 2008 ▶); molecular graphics: *SHELXTL* (Bruker, 2000 ▶); software used to prepare material for publication: *SHELXTL*.

## Supplementary Material

Crystal structure: contains datablocks I, global. DOI: 10.1107/S1600536807068638/bq2059sup1.cif

Structure factors: contains datablocks I. DOI: 10.1107/S1600536807068638/bq2059Isup2.hkl

Additional supplementary materials:  crystallographic information; 3D view; checkCIF report

## Figures and Tables

**Table 1:** Hydrogen-bond geometry (Å, °)

*D*—H⋯*A*	*D*—H	H⋯*A*	*D*⋯*A*	*D*—H⋯*A*
N1*D*—H1*DA*⋯O13*A*^i^	0.89	2.13	2.987 (9)	161
N1*D*—H1*DA*⋯O14*B*^i^	0.89	2.15	2.99 (2)	158
N1*D*—H1*DA*⋯O11*B*^i^	0.89	2.28	2.99 (2)	137
N1*D*—H1*DA*⋯O12*A*^i^	0.89	2.49	3.227 (11)	140
N1*D*—H1*DB*⋯O3	0.89	1.84	2.706 (5)	164
N1*D*—H1*DC*⋯O3^i^	0.89	1.96	2.846 (6)	174
N3*D*—H3*DA*⋯O2^ii^	0.89	2.15	2.842 (5)	134
N3*D*—H3*DA*⋯O13*B*^iii^	0.89	2.22	2.753 (17)	118
N3*D*—H3*DA*⋯O14*A*^iii^	0.89	2.44	3.042 (9)	125
N3*D*—H3*DB*⋯O11*A*	0.89	2.00	2.859 (10)	163
N3*D*—H3*DB*⋯O11*B*	0.89	2.58	3.17 (2)	125
N3*D*—H3*DC*⋯O2	0.89	1.90	2.755 (5)	162
C2*A*—H2*AA*⋯O32*B*^iv^	0.93	2.57	3.422 (6)	152
C5*A*—H5*AA*⋯O11*A*^iii^	0.93	2.56	3.262 (9)	133

Symmetry codes: (i) 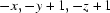; (ii) 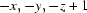; (iii) 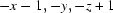; (iv) 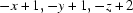.

**Fig. 1.**
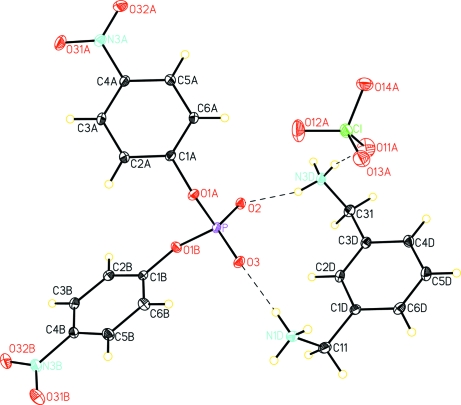
The title compound with numbering scheme used. Hydrogen bonding interactions shown as dotted lines. Ellipsoids are drawn at the 20% probability level.

**Fig. 2.**
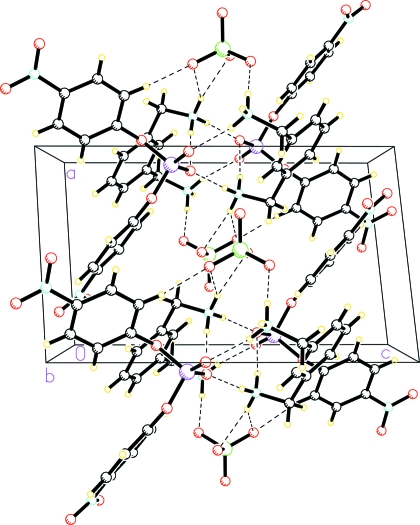
The packing arrangement viewed down the b axis showing the N—H···O and C—H···O interactions (dashed bonds).

## supplementary crystallographic information

### Comment

Base pairing association of biological molecules through hydrogen bonding is central in molecular recognition and attachment of substrates and drugs at specific sites on proteins and the pairing of nucleotides on DNA strands are important phenomena.

The title compound was formed by the reaction of α,α-bis-*m*-xylenediammine and sodium bis-*p*-nitrophenylphosphate in the presence of Zn(ClO_4_).6H_2_O in methanol solution in an effort to study the catalytic activity of the Zn(II) complex of *m*-xylenediamine. The two amine groups on a *meta*-xylenediamine molecule are each protonated and the two ammonium groups are hydrogen bonded to two O atoms of the phosphate anion which acts as a 1,3-bridge at N_amine_—O distances of 2.706 (5) Å and 2.755 (5) Å (Fig. 1.). In addition there are weaker intermolecular interactions with adjoining phosphate (2.842 (5) to 2.846 (6) Å) O atoms of adjoining anions. In the unit cell, the ammonium groups on two *m*-xylenediammonium cations are arranged matched face to face and each pair is doubly intermolecular bridged by two perchlorate ions through hydrogen bonding at N_amine_—O_phosphate_ distances ranging from 2.847 (5) Å to 3.183 (11) Å. The source of H^+^ for the protonation of the amine groups is likely to be the hydrolysis by the aquated Zn^2+^ consistent with the acidic behavior zinc-bound water molecules of the [Zn—OH^2^]^2+^ moiety especially with the assistance of the basic amine groups (Gultneh *et al.*, 1996, Gultneh *et al.*, 1999).

### Experimental

The title compound was formed by the reaction of α,α-bis-*m*-xylenediammine and sodium bis-*p*-nitrophenylphosphate in the presence of Zn(ClO_4_).6H_2_O in a methanol solution. Crystals of the diammonium-phosphate salt crystallized out of the reaction mixture.

### Refinement

The perchlorate O atoms was idealized over two conformations with occupancies of 0.726 (14) and 0.274 (14). The H atoms were idealized with an N—H distance of 0.89 and C—H distances of 0.93 (aromatic C—H), 0.96 (CH_3_), and 0.97 (CH_2_) Å and *U*_iso_(H) = 1.2*U*_eq_(C) (1.5*U*_eq_(C) for the CH_3_ protons). The CH_3_ and NH_3_ protons were allowed to rotate about the C—C and C—N axes, respectively.

### Crystal data

**Table:**

C_8_H_14_N_2_^2+^·C_12_H_8_N_2_O_8_P^–^·ClO_4_^–^	*Z* = 2
*M**_r_* = 576.84	*F*_000_ = 596
Triclinic, *P*1	*D*_x_ = 1.526 Mg m^−^^3^
Hall symbol: -P 1	Mo *K*α radiation λ = 0.71073 Å
*a* = 8.337 (2) Å	Cell parameters from 55 reflections
*b* = 11.623 (3) Å	θ = 2.5–21.5º
*c* = 13.535 (3) Å	µ = 0.29 mm^−^^1^
α = 91.22 (1)º	*T* = 293 (2) K
β = 94.32 (1)º	Prism, pale yellow
γ = 106.06 (1)º	0.35 × 0.29 × 0.17 mm
*V* = 1255.6 (5) Å^3^	

### Data collection

**Table:**

Bruker P4S diffractometer	*R*_int_ = 0.026
Radiation source: fine-focus sealed tube	θ_max_ = 23.0º
Monochromator: graphite	θ_min_ = 2.3º
*T* = 293(2) K	*h* = 0→8
ω scans	*k* = −12→12
Absorption correction: ψ scan(North *et al.*, 1968)	*l* = −14→14
*T*_min_ = 0.876, *T*_max_ = 0.941	3 standard reflections
3673 measured reflections	every 97 reflections
3388 independent reflections	intensity decay: <2%
2390 reflections with *I* > 2σ(*I*)	

### Refinement

**Table:**

Refinement on *F*^2^	Secondary atom site location: difference Fourier map
Least-squares matrix: full	Hydrogen site location: inferred from neighbouring sites
*R*[*F*^2^ > 2σ(*F*^2^)] = 0.058	H-atom parameters constrained
*wR*(*F*^2^) = 0.154	*w* = 1/[σ^2^(*F*_o_^2^) + (0.0642*P*)^2^ + 1.9482*P*] where *P* = (*F*_o_^2^ + 2*F*_c_^2^)/3
*S* = 1.02	(Δ/σ)_max_ < 0.001
3388 reflections	Δρ_max_ = 0.40 e Å^−^^3^
383 parameters	Δρ_min_ = −0.28 e Å^−^^3^
92 restraints	Extinction correction: none
Primary atom site location: structure-invariant direct methods	

### Special details

**Table:**

Geometry. All e.s.d.'s (except the e.s.d. in the dihedral angle between two l.s. planes) are estimated using the full covariance matrix. The cell e.s.d.'s are taken into account individually in the estimation of e.s.d.'s in distances, angles and torsion angles; correlations between e.s.d.'s in cell parameters are only used when they are defined by crystal symmetry. An approximate (isotropic) treatment of cell e.s.d.'s is used for estimating e.s.d.'s involving l.s. planes.
Refinement. Refinement of *F*^2^ against ALL reflections. The weighted *R*-factor *wR* and goodness of fit *S* are based on *F*^2^, conventional *R*-factors *R* are based on *F*, with *F* set to zero for negative *F*^2^. The threshold expression of *F*^2^ > σ(*F*^2^) is used only for calculating *R*-factors(gt) *etc*. and is not relevant to the choice of reflections for refinement. *R*-factors based on *F*^2^ are statistically about twice as large as those based on *F*, and *R*- factors based on ALL data will be even larger.

### Fractional atomic coordinates and isotropic or equivalent isotropic displacement parameters (Å^2^)

**Table:**

	*x*	*y*	*z*	*U*_iso_*/*U*_eq_	Occ. (<1)
Cl	−0.5118 (2)	0.19712 (12)	0.45260 (11)	0.0662 (5)	
P	0.06849 (17)	0.26311 (10)	0.63110 (8)	0.0399 (4)	
O1A	−0.0552 (4)	0.2737 (2)	0.7132 (2)	0.0449 (8)	
O1B	0.2361 (4)	0.2578 (3)	0.6952 (2)	0.0488 (9)	
O2	0.0121 (4)	0.1467 (3)	0.5758 (2)	0.0463 (9)	
O3	0.0908 (4)	0.3765 (3)	0.5791 (2)	0.0513 (9)	
O31A	−0.2691 (6)	−0.0535 (4)	1.0666 (3)	0.0811 (13)	
O32A	−0.4735 (6)	−0.1328 (4)	0.9593 (3)	0.0762 (12)	
O31B	0.7414 (6)	0.6944 (4)	0.9310 (3)	0.0884 (14)	
O32B	0.8000 (6)	0.5482 (4)	1.0050 (3)	0.0906 (15)	
O11A	−0.4392 (10)	0.1435 (9)	0.3755 (6)	0.116 (3)	0.726 (14)
O12A	−0.4323 (16)	0.1876 (9)	0.5441 (6)	0.128 (3)	0.726 (14)
O13A	−0.4991 (12)	0.3164 (6)	0.4320 (8)	0.099 (3)	0.726 (14)
O14A	−0.6843 (9)	0.1299 (7)	0.4453 (7)	0.088 (2)	0.726 (14)
O11B	−0.358 (2)	0.201 (2)	0.5091 (19)	0.113 (6)	0.274 (14)
O12B	−0.480 (3)	0.206 (3)	0.3531 (11)	0.137 (6)	0.274 (14)
O13B	−0.647 (3)	0.1098 (18)	0.4744 (19)	0.103 (6)	0.274 (14)
O14B	−0.542 (2)	0.3121 (14)	0.4838 (17)	0.076 (5)	0.274 (14)
N3A	−0.3393 (7)	−0.0609 (4)	0.9838 (3)	0.0567 (12)	
N3B	0.7181 (6)	0.5890 (5)	0.9438 (3)	0.0639 (13)	
N1D	0.1990 (6)	0.5391 (4)	0.4413 (3)	0.0585 (12)	
H1DA	0.2853	0.5961	0.4703	0.088*	
H1DB	0.1732	0.4773	0.4806	0.088*	
H1DC	0.1112	0.5680	0.4308	0.088*	
N3D	−0.1619 (6)	0.0440 (3)	0.4001 (3)	0.0560 (12)	
H3DA	−0.1761	−0.0325	0.4142	0.084*	
H3DB	−0.2612	0.0589	0.3932	0.084*	
H3DC	−0.0982	0.0909	0.4491	0.084*	
C1A	−0.1208 (6)	0.1835 (4)	0.7777 (3)	0.0402 (12)	
C2A	−0.0489 (7)	0.1960 (4)	0.8735 (3)	0.0542 (14)	
H2AA	0.0459	0.2588	0.8925	0.065*	
C3A	−0.1197 (7)	0.1140 (5)	0.9407 (3)	0.0565 (15)	
H3AA	−0.0734	0.1206	1.0060	0.068*	
C4A	−0.2586 (6)	0.0227 (4)	0.9105 (3)	0.0444 (12)	
C5A	−0.3275 (7)	0.0084 (5)	0.8141 (4)	0.0555 (14)	
H5AA	−0.4199	−0.0559	0.7946	0.067*	
C6A	−0.2572 (7)	0.0915 (4)	0.7463 (3)	0.0526 (14)	
H6AA	−0.3023	0.0844	0.6808	0.063*	
C1B	0.3478 (6)	0.3450 (4)	0.7569 (3)	0.0420 (12)	
C2B	0.4513 (7)	0.3036 (5)	0.8225 (4)	0.0570 (15)	
H2BA	0.4394	0.2218	0.8249	0.068*	
C3B	0.5722 (7)	0.3825 (5)	0.8845 (4)	0.0601 (15)	
H3BA	0.6428	0.3550	0.9287	0.072*	
C4B	0.5863 (6)	0.5025 (5)	0.8797 (3)	0.0508 (13)	
C5B	0.4850 (7)	0.5455 (5)	0.8148 (4)	0.0555 (14)	
H5BA	0.4985	0.6275	0.8122	0.067*	
C6B	0.3629 (7)	0.4661 (4)	0.7533 (4)	0.0523 (13)	
H6BA	0.2915	0.4939	0.7099	0.063*	
C1D	0.1025 (6)	0.4028 (4)	0.2941 (3)	0.0462 (13)	
C2D	0.0742 (7)	0.2853 (4)	0.3231 (3)	0.0480 (13)	
H2DA	0.1425	0.2678	0.3748	0.058*	
C3D	−0.0540 (6)	0.1944 (4)	0.2763 (3)	0.0452 (12)	
C4D	−0.1541 (7)	0.2212 (4)	0.1995 (3)	0.0513 (13)	
H4DA	−0.2411	0.1607	0.1675	0.062*	
C5D	−0.1255 (7)	0.3376 (5)	0.1700 (4)	0.0585 (15)	
H5DA	−0.1931	0.3552	0.1180	0.070*	
C6D	0.0016 (7)	0.4268 (4)	0.2168 (4)	0.0523 (14)	
H6DA	0.0202	0.5049	0.1961	0.063*	
C11	0.2443 (7)	0.4993 (5)	0.3462 (4)	0.0593 (15)	
H11A	0.3413	0.4692	0.3579	0.071*	
H11B	0.2744	0.5672	0.3041	0.071*	
C31	−0.0792 (7)	0.0681 (4)	0.3064 (4)	0.0561 (15)	
H31A	−0.1470	0.0142	0.2539	0.067*	
H31B	0.0286	0.0515	0.3145	0.067*	

### Atomic displacement parameters (Å^2^)

**Table:**

	*U*^11^	*U*^22^	*U*^33^	*U*^12^	*U*^13^	*U*^23^
Cl	0.0684 (12)	0.0577 (9)	0.0649 (9)	0.0092 (8)	−0.0107 (8)	0.0023 (7)
P	0.0529 (9)	0.0357 (7)	0.0278 (6)	0.0075 (6)	0.0011 (6)	0.0040 (5)
O1A	0.060 (2)	0.0365 (17)	0.0373 (17)	0.0105 (16)	0.0115 (16)	0.0053 (14)
O1B	0.049 (2)	0.0420 (18)	0.0501 (19)	0.0059 (16)	−0.0065 (16)	0.0006 (15)
O2	0.061 (2)	0.0393 (18)	0.0357 (17)	0.0107 (16)	0.0000 (16)	−0.0021 (14)
O3	0.072 (3)	0.0381 (18)	0.0417 (18)	0.0107 (17)	0.0052 (17)	0.0120 (14)
O31A	0.112 (4)	0.079 (3)	0.043 (2)	0.011 (2)	0.006 (2)	0.021 (2)
O32A	0.067 (3)	0.071 (3)	0.081 (3)	0.000 (2)	0.012 (2)	0.027 (2)
O31B	0.090 (4)	0.068 (3)	0.094 (3)	0.007 (3)	−0.013 (3)	−0.028 (2)
O32B	0.093 (4)	0.097 (3)	0.062 (3)	0.002 (3)	−0.026 (3)	−0.001 (2)
O11A	0.109 (5)	0.151 (6)	0.107 (5)	0.067 (4)	0.022 (4)	−0.012 (4)
O12A	0.153 (7)	0.141 (5)	0.087 (5)	0.051 (5)	−0.046 (5)	0.012 (4)
O13A	0.100 (5)	0.063 (4)	0.119 (6)	0.007 (3)	−0.017 (5)	0.021 (4)
O14A	0.065 (4)	0.077 (4)	0.113 (5)	0.003 (3)	0.004 (4)	0.030 (3)
O11B	0.111 (9)	0.123 (8)	0.112 (9)	0.052 (7)	−0.023 (7)	0.003 (7)
O12B	0.139 (8)	0.159 (9)	0.114 (8)	0.039 (7)	0.031 (7)	−0.001 (7)
O13B	0.087 (8)	0.091 (8)	0.118 (9)	0.002 (6)	0.016 (7)	0.033 (7)
O14B	0.078 (7)	0.059 (6)	0.090 (8)	0.019 (6)	0.002 (7)	−0.004 (6)
N3A	0.071 (4)	0.048 (3)	0.052 (3)	0.015 (3)	0.013 (3)	0.011 (2)
N3B	0.056 (3)	0.076 (4)	0.050 (3)	0.004 (3)	0.002 (2)	−0.011 (3)
N1D	0.062 (3)	0.048 (2)	0.055 (3)	−0.003 (2)	0.008 (2)	0.006 (2)
N3D	0.077 (3)	0.040 (2)	0.043 (2)	0.007 (2)	−0.010 (2)	0.0052 (18)
C1A	0.050 (3)	0.036 (3)	0.033 (2)	0.008 (2)	0.009 (2)	0.002 (2)
C2A	0.059 (4)	0.054 (3)	0.037 (3)	−0.004 (3)	−0.002 (3)	−0.004 (2)
C3A	0.067 (4)	0.064 (3)	0.031 (3)	0.005 (3)	−0.002 (3)	0.007 (2)
C4A	0.051 (3)	0.046 (3)	0.035 (3)	0.011 (3)	0.006 (2)	0.007 (2)
C5A	0.055 (4)	0.053 (3)	0.045 (3)	−0.005 (3)	−0.002 (3)	0.001 (2)
C6A	0.057 (4)	0.056 (3)	0.034 (3)	−0.001 (3)	−0.003 (2)	0.003 (2)
C1B	0.042 (3)	0.047 (3)	0.032 (2)	0.002 (2)	0.007 (2)	0.005 (2)
C2B	0.061 (4)	0.052 (3)	0.050 (3)	0.002 (3)	0.000 (3)	0.017 (3)
C3B	0.060 (4)	0.071 (4)	0.042 (3)	0.008 (3)	−0.006 (3)	0.016 (3)
C4B	0.044 (3)	0.064 (4)	0.035 (3)	0.001 (3)	0.002 (2)	−0.004 (2)
C5B	0.057 (4)	0.050 (3)	0.054 (3)	0.007 (3)	0.003 (3)	−0.010 (3)
C6B	0.048 (4)	0.052 (3)	0.055 (3)	0.013 (3)	−0.001 (3)	−0.003 (2)
C1D	0.052 (4)	0.042 (3)	0.040 (3)	0.004 (2)	0.008 (2)	0.003 (2)
C2D	0.061 (4)	0.049 (3)	0.036 (3)	0.021 (3)	0.000 (2)	0.006 (2)
C3D	0.056 (4)	0.044 (3)	0.038 (3)	0.017 (3)	0.006 (2)	0.000 (2)
C4D	0.057 (4)	0.051 (3)	0.042 (3)	0.011 (3)	−0.004 (3)	−0.004 (2)
C5D	0.069 (4)	0.059 (3)	0.050 (3)	0.026 (3)	−0.010 (3)	0.005 (3)
C6D	0.069 (4)	0.044 (3)	0.047 (3)	0.018 (3)	0.012 (3)	0.011 (2)
C11	0.065 (4)	0.055 (3)	0.053 (3)	0.005 (3)	0.014 (3)	0.007 (3)
C31	0.081 (4)	0.040 (3)	0.045 (3)	0.013 (3)	0.005 (3)	−0.001 (2)

### Geometric parameters (Å, °)

**Table:**

Cl—O13B	1.347 (14)	C3A—C4A	1.366 (7)
Cl—O12A	1.381 (6)	C3A—H3AA	0.9300
Cl—O12B	1.392 (14)	C4A—C5A	1.373 (6)
Cl—O13A	1.397 (6)	C5A—C6A	1.387 (7)
Cl—O14A	1.429 (7)	C5A—H5AA	0.9300
Cl—O11B	1.430 (14)	C6A—H6AA	0.9300
Cl—O11A	1.456 (7)	C1B—C2B	1.377 (7)
Cl—O14B	1.486 (13)	C1B—C6B	1.381 (7)
P—O2	1.471 (3)	C2B—C3B	1.375 (7)
P—O3	1.480 (3)	C2B—H2BA	0.9300
P—O1A	1.597 (3)	C3B—C4B	1.370 (7)
P—O1B	1.605 (3)	C3B—H3BA	0.9300
O1A—C1A	1.398 (5)	C4B—C5B	1.370 (7)
O1B—C1B	1.385 (5)	C5B—C6B	1.378 (7)
O31A—N3A	1.214 (5)	C5B—H5BA	0.9300
O32A—N3A	1.215 (5)	C6B—H6BA	0.9300
O31B—N3B	1.204 (6)	C1D—C6D	1.374 (7)
O32B—N3B	1.220 (6)	C1D—C2D	1.391 (6)
N3A—C4A	1.469 (6)	C1D—C11	1.504 (7)
N3B—C4B	1.476 (6)	C2D—C3D	1.380 (7)
N1D—C11	1.469 (6)	C2D—H2DA	0.9300
N1D—H1DA	0.8900	C3D—C4D	1.379 (7)
N1D—H1DB	0.8900	C3D—C31	1.494 (6)
N1D—H1DC	0.8900	C4D—C5D	1.380 (7)
N3D—C31	1.484 (6)	C4D—H4DA	0.9300
N3D—H3DA	0.8900	C5D—C6D	1.365 (7)
N3D—H3DB	0.8900	C5D—H5DA	0.9300
N3D—H3DC	0.8900	C6D—H6DA	0.9300
C1A—C6A	1.361 (6)	C11—H11A	0.9700
C1A—C2A	1.374 (6)	C11—H11B	0.9700
C2A—C3A	1.375 (7)	C31—H31A	0.9700
C2A—H2AA	0.9300	C31—H31B	0.9700
			
O13B—Cl—O12A	89.9 (11)	C2A—C3A—H3AA	120.3
O13B—Cl—O12B	116.6 (13)	C3A—C4A—C5A	121.8 (4)
O12A—Cl—O12B	140.3 (11)	C3A—C4A—N3A	119.1 (4)
O13B—Cl—O13A	129.2 (12)	C5A—C4A—N3A	119.0 (5)
O12A—Cl—O13A	111.7 (6)	C4A—C5A—C6A	119.0 (5)
O12B—Cl—O13A	75.2 (11)	C4A—C5A—H5AA	120.5
O12A—Cl—O14A	111.7 (5)	C6A—C5A—H5AA	120.5
O12B—Cl—O14A	101.4 (11)	C1A—C6A—C5A	118.7 (4)
O13A—Cl—O14A	109.4 (5)	C1A—C6A—H6AA	120.7
O13B—Cl—O11B	115.6 (12)	C5A—C6A—H6AA	120.7
O12B—Cl—O11B	108.0 (11)	C2B—C1B—C6B	120.5 (5)
O13A—Cl—O11B	104.9 (11)	C2B—C1B—O1B	115.2 (4)
O14A—Cl—O11B	139.3 (11)	C6B—C1B—O1B	124.3 (4)
O13B—Cl—O11A	104.8 (12)	C3B—C2B—C1B	120.4 (5)
O12A—Cl—O11A	109.8 (5)	C3B—C2B—H2BA	119.8
O13A—Cl—O11A	109.4 (5)	C1B—C2B—H2BA	119.8
O14A—Cl—O11A	104.6 (5)	C4B—C3B—C2B	118.5 (5)
O11B—Cl—O11A	83.4 (10)	C4B—C3B—H3BA	120.8
O13B—Cl—O14B	106.2 (12)	C2B—C3B—H3BA	120.8
O12A—Cl—O14B	92.2 (9)	C5B—C4B—C3B	122.0 (5)
O12B—Cl—O14B	106.5 (11)	C5B—C4B—N3B	118.6 (5)
O14A—Cl—O14B	95.1 (9)	C3B—C4B—N3B	119.4 (5)
O11B—Cl—O14B	102.6 (10)	C4B—C5B—C6B	119.4 (5)
O11A—Cl—O14B	141.7 (8)	C4B—C5B—H5BA	120.3
O2—P—O3	120.85 (18)	C6B—C5B—H5BA	120.3
O2—P—O1A	111.69 (18)	C5B—C6B—C1B	119.2 (5)
O3—P—O1A	103.26 (18)	C5B—C6B—H6BA	120.4
O2—P—O1B	104.05 (18)	C1B—C6B—H6BA	120.4
O3—P—O1B	112.33 (19)	C6D—C1D—C2D	118.8 (5)
O1A—P—O1B	103.54 (17)	C6D—C1D—C11	121.9 (4)
C1A—O1A—P	124.4 (3)	C2D—C1D—C11	119.3 (5)
C1B—O1B—P	129.7 (3)	C3D—C2D—C1D	120.9 (4)
O31A—N3A—O32A	123.2 (5)	C3D—C2D—H2DA	119.6
O31A—N3A—C4A	118.1 (5)	C1D—C2D—H2DA	119.6
O32A—N3A—C4A	118.7 (4)	C4D—C3D—C2D	119.2 (4)
O31B—N3B—O32B	124.1 (5)	C4D—C3D—C31	120.7 (5)
O31B—N3B—C4B	118.6 (5)	C2D—C3D—C31	120.1 (4)
O32B—N3B—C4B	117.2 (5)	C3D—C4D—C5D	120.1 (5)
C11—N1D—H1DA	109.5	C3D—C4D—H4DA	120.0
C11—N1D—H1DB	109.5	C5D—C4D—H4DA	119.9
H1DA—N1D—H1DB	109.5	C6D—C5D—C4D	120.3 (5)
C11—N1D—H1DC	109.5	C6D—C5D—H5DA	119.9
H1DA—N1D—H1DC	109.5	C4D—C5D—H5DA	119.9
H1DB—N1D—H1DC	109.5	C5D—C6D—C1D	120.8 (5)
C31—N3D—H3DA	109.5	C5D—C6D—H6DA	119.6
C31—N3D—H3DB	109.5	C1D—C6D—H6DA	119.6
H3DA—N3D—H3DB	109.5	N1D—C11—C1D	111.7 (4)
C31—N3D—H3DC	109.5	N1D—C11—H11A	109.3
H3DA—N3D—H3DC	109.5	C1D—C11—H11A	109.3
H3DB—N3D—H3DC	109.5	N1D—C11—H11B	109.3
C6A—C1A—C2A	122.4 (4)	C1D—C11—H11B	109.3
C6A—C1A—O1A	119.9 (4)	H11A—C11—H11B	108.0
C2A—C1A—O1A	117.5 (4)	N3D—C31—C3D	112.9 (4)
C1A—C2A—C3A	118.7 (5)	N3D—C31—H31A	109.0
C1A—C2A—H2AA	120.6	C3D—C31—H31A	109.0
C3A—C2A—H2AA	120.6	N3D—C31—H31B	109.0
C4A—C3A—C2A	119.3 (4)	C3D—C31—H31B	109.0
C4A—C3A—H3AA	120.3	H31A—C31—H31B	107.8
			
O2—P—O1A—C1A	44.8 (4)	C1B—C2B—C3B—C4B	0.3 (8)
O3—P—O1A—C1A	176.1 (3)	C2B—C3B—C4B—C5B	−0.6 (8)
O1B—P—O1A—C1A	−66.6 (4)	C2B—C3B—C4B—N3B	−178.4 (5)
O2—P—O1B—C1B	175.9 (4)	O31B—N3B—C4B—C5B	−6.2 (7)
O3—P—O1B—C1B	43.5 (4)	O32B—N3B—C4B—C5B	175.8 (5)
O1A—P—O1B—C1B	−67.2 (4)	O31B—N3B—C4B—C3B	171.7 (5)
P—O1A—C1A—C6A	−81.8 (5)	O32B—N3B—C4B—C3B	−6.3 (7)
P—O1A—C1A—C2A	101.9 (5)	C3B—C4B—C5B—C6B	1.1 (8)
C6A—C1A—C2A—C3A	−1.5 (8)	N3B—C4B—C5B—C6B	179.0 (5)
O1A—C1A—C2A—C3A	174.7 (5)	C4B—C5B—C6B—C1B	−1.5 (8)
C1A—C2A—C3A—C4A	0.1 (8)	C2B—C1B—C6B—C5B	1.3 (8)
C2A—C3A—C4A—C5A	1.8 (9)	O1B—C1B—C6B—C5B	−176.5 (4)
C2A—C3A—C4A—N3A	−176.7 (5)	C6D—C1D—C2D—C3D	−0.8 (7)
O31A—N3A—C4A—C3A	−7.7 (7)	C11—C1D—C2D—C3D	−179.5 (5)
O32A—N3A—C4A—C3A	171.3 (5)	C1D—C2D—C3D—C4D	0.3 (7)
O31A—N3A—C4A—C5A	173.8 (5)	C1D—C2D—C3D—C31	178.1 (5)
O32A—N3A—C4A—C5A	−7.1 (7)	C2D—C3D—C4D—C5D	0.2 (8)
C3A—C4A—C5A—C6A	−2.2 (8)	C31—C3D—C4D—C5D	−177.6 (5)
N3A—C4A—C5A—C6A	176.2 (5)	C3D—C4D—C5D—C6D	−0.2 (8)
C2A—C1A—C6A—C5A	1.1 (8)	C4D—C5D—C6D—C1D	−0.4 (8)
O1A—C1A—C6A—C5A	−175.0 (5)	C2D—C1D—C6D—C5D	0.8 (8)
C4A—C5A—C6A—C1A	0.8 (8)	C11—C1D—C6D—C5D	179.5 (5)
P—O1B—C1B—C2B	162.3 (4)	C6D—C1D—C11—N1D	101.4 (5)
P—O1B—C1B—C6B	−19.9 (7)	C2D—C1D—C11—N1D	−80.0 (6)
C6B—C1B—C2B—C3B	−0.7 (8)	C4D—C3D—C31—N3D	−105.0 (5)
O1B—C1B—C2B—C3B	177.2 (5)	C2D—C3D—C31—N3D	77.3 (6)

### Hydrogen-bond geometry (Å, °)

**Table:**

*D*—H···*A*	*D*—H	H···*A*	*D*···*A*	*D*—H···*A*
N1D—H1DA···O13A^i^	0.89	2.13	2.987 (9)	161
N1D—H1DA···O14B^i^	0.89	2.15	2.99 (2)	158
N1D—H1DA···O11B^i^	0.89	2.28	2.99 (2)	137
N1D—H1DA···O12A^i^	0.89	2.49	3.227 (11)	140
N1D—H1DB···O3	0.89	1.84	2.706 (5)	164
N1D—H1DC···O3^i^	0.89	1.96	2.846 (6)	174
N3D—H3DA···O2^ii^	0.89	2.15	2.842 (5)	134
N3D—H3DA···O13B^iii^	0.89	2.22	2.753 (17)	118
N3D—H3DA···O14A^iii^	0.89	2.44	3.042 (9)	125
N3D—H3DB···O11A	0.89	2.00	2.859 (10)	163
N3D—H3DB···O11B	0.89	2.58	3.17 (2)	125
N3D—H3DC···O2	0.89	1.90	2.755 (5)	162
C2A—H2AA···O32B^iv^	0.93	2.57	3.422 (6)	152
C5A—H5AA···O11A^iii^	0.93	2.56	3.262 (9)	133

Symmetry codes: (i) −*x*, −*y*+1, −*z*+1; (ii) −*x*, −*y*, −*z*+1; (iii) −*x*−1, −*y*, −*z*+1; (iv) −*x*+1, −*y*+1, −*z*+2.
